# Perceived risk of infection and death from COVID-19 among community members of low- and middle-income countries: A cross-sectional study

**DOI:** 10.12688/f1000research.109575.1

**Published:** 2022-03-22

**Authors:** Mahir Gachabayov, Khan Sharun, Daniel M. Felsenreich, Firzan Nainu, Samsul Anwar, Amanda Yufika, Youdiil Ophinni, Chika Yamada, Marhami Fahriani, Milda Husnah, Rawan Raad, Namareg ME. Khiri, Rashed YA. Abdalla, Rashed Y. Adam, Mohajer IH. Ismaeil, Asma Y. Ismail, Wajdi Kacem, Zeineb Teyeb, Khaoula Aloui, Montacer Hafsi, Manel Ferjani, Nasrine Ben Hadj Dahman, Dalia A. Deeb, Dina Emad, Kirellos Said Abbas, Fatma A. Monib, Farah S. Sami, Subramaniam Ramanarayanan, Suhrud Panchawagh, Sunil Anandu, Md Ariful Haque, Lirane ED. Ferreto, María FC. Briones, Rocío BI. Morales, Sebastián Lazcano-Díaz, José TO. Aburto, Jorge ET. Rojas, Emmanuel O. Balogun, Hendrix I. Kusuma, Cut Meurah Yeni, Niken Asri Utami, Seyi S. Enitan, Akele R. Yomi, Abiodun Durosinmi, Esther N. Adejumo, Eyiuche D. Ezigbo, Elham Babadi, Edris Kakemam, Irfan Ullah, Najma I. Malik, Francesco Rosiello, Talha B. Emran, Eva Imelda, Guilherme W. Wendt, Morteza Arab-Zozani, Kuldeep Dhama, Mudatsir Mudatsir, Harapan Harapan

**Affiliations:** 1 Department of Abdominal Surgery, Vladimir City Emergency Hospital, Vladimir, 600014, Russian Federation; 2 Division of Surgery, ICAR-Indian Veterinary Research Institute, Izatnagar, Bareilly, Uttar Pradesh, 243122, India; 3 Division of General Surgery, Department of Surgery, Medical University of Vienna, Vienna, 1090, Austria; 4 Department of Pharmacy, Faculty of Pharmacy, Hasanuddin University, Makassar, 90245, Indonesia; 5 Department of Statistics, Faculty of Mathematics and Natural Sciences, Universitas Syiah Kuala, Banda Aceh, 23111, Indonesia; 6 Department of Family Medicine, School of Medicine, Universitas Syiah Kuala, Banda Aceh, 23111, Indonesia; 7 Ragon Institute of MGH, MIT and Harvard, Cambridge, 02139, USA; 8 Laboratory of Host Defense, WPI Immunology Frontier Research Center (IFReC), Osaka University, Osaka, 565-0874, Japan; 9 Department of Environmental Coexistence, Center for Southeast Asian Studies, Kyoto University, Kyoto, 606-8304, Japan; 10 Medical Research Unit, School of Medicine, Universitas Syiah Kuala, Banda Aceh, 23111, Indonesia; 11 Master Program of Biology, Faculty of Mathematics and Natural Sciences, Universitas Syiah Kuala, BAnda Aceh, 23111, Indonesia; 12 Faculty of Medicine and General Surgery, Sudan University of Science and Technology, Khartoum, 407, Sudan; 13 Faculty of Medicine, University of Khartoum, Omdurman, 11111, Sudan; 14 Faculty of Medicine, University of Bahri, Khartoum, 11111, Sudan; 15 Omdurman Teaching Hospital, Khartoum, 00000, Sudan; 16 Faculty of Medicine, Alzaiem Alazhari University, Khartoum, 13311, Sudan; 17 Department of Internal Medicine, Faculty of Medicine, Sudan International University, Khartoum, 12769, Sudan; 18 Department of Emergency Medicine, Faculty of Medicine of Tunis, University Tunis El Manar, Tunis, 2074, Tunisia; 19 Department of Internal Medicine, Faculty of Medicine of Tunis, University of Tunis El Manar, Tunis, 2074, Tunisia; 20 Faculty of Medicine of Tunis, University of Tunis El Manar, Tunis, 2074, Tunisia; 21 Faculty of Dental Medicine, University of Monastir, Monastir, 4180, Tunisia; 22 Faculty of Medicine, Zagazig University, El-sharkia, 44519, Egypt; 23 Faculty of Medicine, Ain Shams University Nasr City, Cairo, 1181, Egypt; 24 Faculty of Medicine, Alexandria University, Alexandria, 21131, Egypt; 25 Faculty of Medicine, Assiut University, Assiut, 71515, Egypt; 26 Department of Public Health Dentistry, Indira Gandhi Institute of Dental Sciences, Nellikuzhi, Kothamangalam, Kerala, 686691, India; 27 Department of General Medicine, Smt. KashibaiNavale Medical College and General Hospital, Pune, Maharashtra, 411041, India; 28 Division of Veterinary Parasitology, ICAR-Indian Veterinary Research Institute, Izatnagar, Bareilly, Uttar Pradesh, 243122, India; 29 Department of Orthopedic Surgery, Yan’an Hospital Affiliated to Kunming Medical University, Kunming, Yunnan, 650000, China; 30 Department of Public Health and Postgraduate Program in Applied Health Sciences, Faculty of Medicine, Western Paraná State University, Francisco Beltrão, 85601-970, Brazil; 31 Faculty of Medicine, University of La Frontera, Temuco, 4781218, Chile; 32 Department of Biochemistry, Faculty of Life Sciences, Ahmadu Bello University, Zaria, Kaduna State, 2222, Nigeria; 33 Department of Biology Education, Faculty of Tarbiyah and Teacher Training, Universitas Islam Negeri Ar-Raniry, Banda Aceh, 23111, Indonesia; 34 Department of Obstetrics and Gynecology, School of Medicine, Universitas Syiah Kuala, Banda Aceh, 23111, Indonesia; 35 Department of Obstetrics and Gynecology, Dr. Zainoel Abidin Hospital, Banda Aceh, 24415, Indonesia; 36 Department of Medical Laboratory Science, Babcock University, Ilishan-Remo, Ogun State, 121103, Nigeria; 37 Afe Babalola University, Ado Ekiti, 360102, Nigeria; 38 State Hospital, Ijebu Ode, Ogun State, 120101, Nigeria; 39 Department of Medical Laboratory Sciences, Faculty of Health Sciences & Technology, University of Nigeria, Nsukka, Enugu State, 40006, Nigeria; 40 Research Fellow, Mayo Clinic, Rochester, 14604, USA; 41 Tabriz Health Services Management Research Center, Tabriz University of Medical Sciences, Tabriz, 516599001, Iran; 42 Department of Internal Medicine, Kabir Medical College, Gandhara University, Peshawar, 25000, Pakistan; 43 Department of Psychology, University of Sargodha, Sargodha, 40100, Pakistan; 44 Department of Public Health and Infectious Disease, Sapienza-University of Rome, Rome, 00185, Italy; 45 Department of Pharmacy, BGC Trust University Bangladesh, Chittagong, 4381, Bangladesh; 46 Department of Ophthalmology, School of Medicine, Universitas Syiah Kuala, Banda Aceh, 23111, Indonesia; 47 Department of Life Sciences, Faculty of Medicine, Western Paraná State University, Francisco Beltrão, 85601-970, Brazil; 48 Social Determinants of Health Research Center, Birjand University of Medical Sciences, Birjand, 97, Iran; 49 Division of Pathology, ICAR-Indian Veterinary Research Institute,, Izatnagar, Bareilly, Uttar Pradesh, 243122, India; 50 Tropical Disease Centre, School of Medicine, Universitas Syiah Kuala, Banda Aceh, 23111, Indonesia; 51 Department of Microbiology, School of Medicine, Universitas Syiah Kuala, Banda Aceh, 23111, Indonesia; 52 Tsunami and Disaster Mitigation Research Centre, Universitas Syiah Kuala, Banda Aceh, 231111, Indonesia

**Keywords:** COVID-19, perceived risk, online cross-sectional study, preventive measure, determinants

## Abstract

**Background: **Risk perceptions of coronavirus disease 2019 (COVID-19) are considered important as they impact community health behaviors. The aim of this study was to determine the perceived risk of infection and death due to COVID-19 and to assess the factors associated with such risk perceptions among community members in low- and middle-income countries (LMICs) in Africa, Asia, and South America.

**Methods: **An online cross-sectional study was conducted in 10 LMICs in Africa, Asia, and South America from February to May 2021. A questionnaire was utilized to assess the perceived risk of infection and death from COVID-19 and its plausible determinants. A logistic regression model was used to identify the factors associated with such risk perceptions.

**Results:** A total of 1,646 responses were included in the analysis of the perceived risk of becoming infected and dying from COVID-19. Our data suggested that 36.4% of participants had a high perceived risk of COVID-19 infection, while only 22.4% had a perceived risk of dying from COVID-19. Being a woman, working in healthcare-related sectors, contracting pulmonary disease, knowing people in the immediate social environment who are or have been infected with COVID-19, as well as seeing or reading about individuals infected with COVID-19 on social media or TV were all associated with a higher perceived risk of becoming infected with COVID-19. In addition, being a woman, elderly, having heart disease and pulmonary disease, knowing people in the immediate social environment who are or have been infected with COVID-19, and seeing or reading about individuals infected with COVID-19 on social media or TV had a higher perceived risk of dying from COVID-19.

**Conclusions:** The perceived risk of infection and death due to COVID-19 are relatively low among respondents; this suggests the need to conduct health campaigns to disseminate knowledge and information on the ongoing pandemic.

## Introduction

The coronavirus disease 2019 (COVID-19), caused by the severe acute respiratory syndrome coronavirus 2 (SARS-CoV-2), has had catastrophic effects on the economy, health, and society ^1^^–^^3^ and is associated with long-term health problem. ^4^^,^^5^ The risk perceptions of COVID-19 are considered important as they influence health behaviors in the community. ^6^ According to one study, accessing the media to obtain information on COVID-19 is linked to an increase in COVID-19 perceived risk and severity. ^7^ In addition, a study found that the willingness to receive COVID-19 vaccination was related to the perceived risk of becoming infected with SARS-CoV-2. ^8^ Therefore, providing reliable information on the risks of infection and possible adverse health outcomes (including death) could directly influence an individual’s risk perceptions. This, in turn, may affect their decisions to select suitable preventive measures. ^9^

Several studies have analyzed risk perceptions during the ongoing pandemic to learn more about their correlates and predictors. ^6^^,^^7^^,^^9^^,^^10^ In general, the findings from these studies suggest that risk perceptions of SARS-CoV-2 infection are predictive of the adoption of different protective behaviors. ^6^^,^^9^ Furthermore, strategic interventions aimed at increasing the risk perceptions of individuals can help promote desired protective behaviors. ^6^ In addition, perceived risks are also considered as important drivers for the adoption of COVID-19 control measures implemented by the government and other responsible agencies. ^10^

Studies that assess risk perceptions and the associated determining factors will provide policymakers with critical information that will help to design population-wide health measures to combat the ongoing pandemic. In addition, long-term COVID-19 risk perception studies, especially in low- and middle-income countries (LMICs), will help to develop efficient preventive strategies within the population. ^9^ The present study was designed to determine the perceived risk of becoming infected and dying as a result of COVID-19, as well as to assess the factors associated with such risk perceptions in the community members of LMICs in Africa, Asia, and South America.

## Methods

### Study design and setting

We conducted an online cross-sectional study ^23^ in 10 LMICs in Africa, Asia, and South America between February and May 2021. The survey link, hosted by SurveyMonkey, was shared on Twitter, Facebook, and WhatsApp. The survey ^22^ consisted of three sections: an introduction page with study information and an informed consent page where respondents had to provide consent to participate, and the main survey, which asked respondents about their demographic background, previous health conditions, perceived risk of infection and death due to COVID-19, and several possible determinants. The survey included an informed consent page that included the benefits of the study, risks and discomforts and information that this study was completely voluntary. All respondents provided consent to participate by clicking “Agree” before the next page could be opened. It took approximately 15 minutes to complete all the questions. Before being utilized in the study, the questions in the questionnaire were evaluated and their validity was confirmed. Most of the questions were adopted from a previous study ^11^ and during the validity assessment, each question was assessed by experts in the fields of virology and public health. Changes were made to the questionnaire based on the validity assessment. No further reliability or validity tests in the field were made. The inclusion criteria to be eligible in this survey were those who were aged over 18 years old and able to read and understand English in the 10 studied countries. We sought a 95% confidence level and a 5% margin of error to recruit the minimal sample size with a conservative assumption that 50% of respondents having good perceived risk of COVID-19. We employed a convenience sampling approach, a non-probability sampling method, to recruit the samples. We received 1,849 responses during the study of which 203 respondents were excluded due to incomplete information.

The Institutional Review Board of Universitas Syiah Kuala & Zainoel Abidin Hospital (129/EA/FK-RSUDZA/2021) approved the study protocol and it was registered with the Indonesian National Health Research and Development Ethics Commission (1171012P).

### Study variables

*Response variable*

The response variable in this study was the perceived risk of COVID-19. To assess the perceived risk, the respondents were asked two questions: (1) “What do you think are the chances that you will get COVID-19 in the next month?” and (2) “What do you think is your risk of dying from COVID-19 if infected?” A slide bar was provided to the respondents, with which they could move the percentage between 0% and 100%. The perceived risk score was then classified into low (a score equal to or less than 50%) and high (a score of more than 50%). Each question was analyzed separately.

*Explanatory variables*

Age, gender, urbanicity, monthly household income in USD, religion, occupation sector (healthcare- and non-healthcare-related), type of occupation, and the presence of COVID-19 comorbidities based on self-reports, such as hypertension, diabetes, heart disease, and pulmonary disease were all included as explanatory variables. In addition, the respondents were asked whether they knew anyone in their immediate social environment who were or had been infected with COVID-19. Respondents were also questioned about their exposure to information (have seen or read) of individuals infected with COVID-19 on TV or social media.

### Statistical analysis

Logistic regression analysis was used to assess the factors associated with the perceived risk of contracting COVID-19 and the factors associated with the perceived risk of dying from COVID-19 if infected. The unadjusted logistic regressions, crude odds ratio (OR), and 95% confidence interval (CI) of each plausible factor were calculated separately in the first step. Factors with p-values of less than 0.25 in the univariate analysis were included in the adjusted analysis, where the adjusted OR (aOR) was calculated. The analyses were conducted using SPSS software version 24 (SPSS Inc., Chicago, IL, USA) (SPSS, RRID:SCR_019096).

## Results

### Demographic characteristics

Table 1 ^22^ presents the characteristics of the 1,646 responses that were analyzed. India and Pakistan had the highest number of respondents. More than half of the participants were between the ages of 21 and 30, and 58% of the total respondents were female. The majority (81%) of the respondents lived in urban areas, and 65% were part of a family with a monthly household income of less than $2,000. Respondents with hypertension, diabetes, heart disease, and pulmonary disease made up 5.9%, 3.5%, 3.3%, and 5.5% of the study participants, respectively. Almost 70% of the respondents knew someone who had been infected with COVID-19 in their immediate social environment, and more than 92.7% had seen or read about individuals infected with COVID-19 on social media or TV.

**Table 1.** Logistic regression showing factors associated with perceived risk of COVID-19 infection (n=1,649).

Variable	n (%)	High perceived risk	Unadjusted		Adjusted	
		n (%)	OR (95% CI)	p-value	OR (95% CI)	p-value
Country						
Pakistan *(R)*	263 (15.9)	52 (19.8)	1		1	
Brazil	107 (6.5)	40 (37.4)	2.42 (1.48 – 3.98)	<0.001	2.32 (1.13 – 4.77)	0.022
Chile	106 (6.4)	23 (21.7)	1.12 (0.65 – 1.95)	0.677	1.40 (0.66 – 2.97)	0.379
Egypt	98 (6.0)	50 (51.0)	4.23 (2.57 – 6.96)	<0.001	2.35 (1.36 – 4.07)	0.002
India	339 (20.6)	149 (44.0)	3.18 (2.19 – 4.61)	<0.001	4.28 (2.33 – 7.86)	<0.001
Iran	141 (8.6)	53 (37.6)	2.44 (1.55 – 3.86)	<0.001	1.30 (0.75 – 2.24)	0.353
Nigeria	161 (9.8)	28 (17.4)	0.85 (0.51 – 1.42)	0.543	1.00 (0.50 – 2.00)	0.995
Bangladesh	131 (7.9)	54 (41.2)	2.85 (1.79 – 4.52)	<0.001	2.86 (1.68 – 4.87)	<0.001
Sudan	174 (10.6)	76 (43.7)	3.15 (2.05 – 4.82)	<0.001	1.61 (0.97 – 2.67)	0.066
Tunisia	129 (7.8)	75 (58.1)	5.64 (3.55 – 8.96)	<0.001	3.66 (2.12 – 6.32)	<0.001
Age group (year)						
<20 *(R)*	280 (17.0)	63 (22.5)	1		1	
21-30	926 (56.2)	373 (40.3)	2.32 (1.70 – 3.17)	<0.001	1.50 (1.03 – 2.18)	0.035
31-40	270 (16.4)	115 (42.6)	2.56 (1.77 – 3.70)	<0.001	1.69 (1.02 – 2.82)	0.043
41-50	119 (7.2)	33 (27.7)	1.32 (0.81 – 2.16)	0.264	0.90 (0.48 – 1.69)	0.745
>51	54 (3.3)	16 (29.6)	1.45 (0.76 – 2.77)	0.261	0.71 (0.31 – 1.63)	0.423
Sex						
Male *(R)*	692 (42.0)	234 (33.8)	1		1	
Female	957 (58.0)	366 (38.2)	1.21 (0.99 – 1.49)	0.065	1.40 (1.10 – 1.78)	0.006
Urbanicity						
Rural *(R)*	314 (19.0)	116 (36.9)	1			
Urban	1335 (81.0)	484 (36.3)	0.97 (0.75 – 1.25)	0.820		
Monthly household income (USD)						
<500 *(R)*	618 (37.5)	228 (36.9)	1		1	
500-999	289 (17.5)	110 (38.1)	1.05 (0.79 – 1.40)	0.734	1.02 (0.74 – 1.40)	0.921
1,000-1,999	192 (11.6)	72 (37.5)	1.03 (0.73 – 1.43)	0.879	0.92 (0.63 – 1.35)	0.665
2,000-2,999	148 (9.0)	63 (42.6)	1.27 (0.88 – 1.83)	0.202	1.29 (0.85 – 1.98)	0.236
3,000-4,999	128 (7.8)	39 (30.5)	0.75 (0.50 – 1.13)	0.169	0.79 (0.50 – 1.26)	0.319
5,000-7,999	100 (6.1)	36 (36.0)	0.96 (0.62 – 1.49)	0.864	1.00 (0.60 – 1.64)	0.984
≥8,000	174 (10.6)	52 (29.9)	0.73 (0.51 – 1.05)	0.088	0.67 (0.43 – 1.05)	0.080
Religion						
Islam *(R)*	915 (55.5)	354 (38.7)	1		1	
Christian/Protestant/Methodist/Lutheran/Baptist	179 (10.9)	46 (25.7)	0.55 (0.38 – 0.79)	0.001	0.65 (0.38 – 1.11)	0.117
Catholic	127 (7.7)	41 (32.3)	0.76 (0.51 – 1.12)	0.164	0.68 (0.37 – 1.25)	0.213
Hindu	239 (14.5)	92 (38.5)	0.99 (0.74 – 1.33)	0.956	0.44 (0.26 – 0.74)	0.002
Atheist or agnostic	87 (5.3)	25 (28.7)	0.64 (0.39 – 1.04)	0.069	0.50 (0.27 – 0.93)	0.028
Others	102 (6.2)	42 (41.2)	1.11 (0.73 – 1.68)	0.625	0.84 (0.49 – 1.43)	0.510
Healthcare-related job						
No *(R)*	908 (55.1)	261 (28.7)	1		1	
Yes	741 (44.9)	339 (45.7)	2.09 (1.71 – 2.56)	<0.001	1.48 (1.16 – 1.89)	0.002
Have hypertension						
No ^a^ *(R)*	1102 (66.8)	404 (36.7)	1			
Yes ^b^	97 (5.9)	34 (35.1)	0.93 (0.60 – 1.44)	0.752		
Do not know	450 (27.3)	162 (36.0)	0.97 (0.77 – 1.22)	0.806		
Have diabetes						
No ^a^ *(R)*	1190 (72.2)	447 (37.6)	1		1	
Yes ^b^	58 (3.5)	19 (32.8)	0.81 (0.46 – 1.42)	0.461	0.79 (0.40 – 1.55)	0.487
Do not know	401 (24.3)	134 (33.4)	0.83 (0.66 – 1.06)	0.136	0.79 (0.56 – 1.13)	0.200
Have heart disease						
No ^a^ *(R)*	1093 (66.3)	395 (36.1)	1		1	
Yes ^b^	55 (3.3)	26 (47.3)	1.58 (0.92 – 2.73)	0.097	1.59 (0.82 – 3.07)	0.166
Do not know	501 (30.4)	179 (35.7)	0.98 (0.79 – 1.23)	0.874	1.07 (0.71 – 1.59)	0.755
Have pulmonary disease						
No ^a^ *(R)*	1044 (63.3)	371 (35.5)	1		1	
Yes ^b^	90 (5.5)	51 (56.7)	2.37 (1.53 – 3.67)	<0.001	2.44 (1.48 – 4.05)	0.001
Do not know	515 (31.2)	178 (34.6)	0.96 (0.77 – 1.20)	0.705	1.22 (0.82 – 1.82)	0.335
Know people in immediate social environment who are or have been infected with COVID-19						
No *(R)*	508 (30.8)	105 (20.7)	1		1	
Yes	1141 (69.2)	495 (43.4)	2.94 (2.30 – 3.76)	<0.001	2.05 (1.55 – 2.70)	<0.001
Have you seen or read about individuals infected with the COVID-19 on social media or TV?						
No *(R)*	121 (7.3)	27 (22.3)	1		1	
Yes	1528 (92.7)	573 (37.5)	2.09 (1.34 – 3.24)	<0.001	1.72 (1.06 – 2.77)	0.027
Occupation						
Self-employed *(R)*	155 (9.4)	42 (27.1)	1		1	
Employed for wages	417 (25.3)	177 (42.4)	1.98 (1.33 – 2.97)	<0.001	2.22 (1.43 – 3.45)	<0.001
Out of work for less 1 year AND more than 1 year	73 (4.4)	23 (31.5)	1.24 (0.67 – 2.27)	0.492	1.49 (0.77 – 2.89)	0.241
Homemaker	34 (2.1)	12 (35.3)	1.47 (0.67 – 3.23)	0.340	1.65 (0.68 – 4.00)	0.272
Student	948 (57.5)	330 (34.8)	1.44 (0.98 – 2.10)	0.061	2.05 (1.31 – 3.22)	0.002
Retired or unable to work	22 (1.3)	16 (72.7)	7.18 (2.63 – 19.56)	<0.001	11.36 (3.81 – 33.87)	<0.001
Has how much your work changed as a result of the COVID-19 pandemic?						
No change and not applicable (not working) *(R)*	1009 (61.2)	349 (34.6)	1		1	
I work more hours	300 (18.2)	126 (42.0)	1.37 (1.05 – 1.78)	0.019	1.17 (0.85 – 1.59)	0.336
I work fewer hours	286 (17.3)	107 (37.4)	1.13 (0.86 – 1.48)	0.378	1.02 (0.75 – 1.39)	0.900
I was let go from my job	54 (3.3)	18 (33.3)	0.95 (0.53 – 1.69)	0.850	0.90 (0.46 – 1.74)	0.753

COVID-19, coronavirus disease 2019; OR, odds ratio; CI, confidence interval; R, reference group.

^a^ Have been tested or examined by a doctor but negative.

^b^ Have been diagnosed by a doctor.

### Perceived risk of becoming infected with COVID-19 and the associated determinants

Of the 1,646 respondents, 36.4% (600/1,646) had a high perceived risk of being infected with COVID-19. The unadjusted logistic regression suggested that the country, age group, religion, type of job, having pulmonary disease, knowing people in the immediate social environment who were or had been infected with COVID-19, seeing or reading about individuals infected with COVID-19 on social media or TV, and type of occupation were associated with a perceived risk of contracting COVID-19 ( Table 1). In the adjusted logistic regression, only country, age group, gender, religion, type of job, those with pulmonary disease, knowing people in the immediate social environment who were or had been infected with COVID-19, seeing or reading about individuals infected with COVID-19 on social media or TV, and type of occupation were associated with a perceived risk of contracting COVID-19.

Females had a greater perceived risk of contracting COVID-19 compared to males (aOR: 1.40; 95% CI: 1.10–1.78, p=0.006) ( Table 1). Those working in healthcare-related sectors had almost 1.48 times higher odds of a higher perceived risk of contracting COVID-19 compared to those working in non-healthcare sectors (aOR: 1.48; 95% CI: 1.16–1.89, p=0.002). Our study also found that people with COVID-19 comorbidities, in particular pulmonary disease, had a higher perceived risk compared to those with no pulmonary disease (aOR: 2.44; 95% CI: 1.48–4.05). Participants who knew someone from their immediate social environment who were or had been infected with COVID-19, as well as those who had seen or read about COVID-19 cases on social media or TV, had 2.05 (95% CI: 1.55–2.70) and 1.72 (95% CI: 1.06–2.77) times higher odds of perceived risk of becoming infected with COVID-19 compared to those who did not know and had never seen or read about COVID-19 cases, respectively. Compared to self-employed participants, those who were employed for wages, students, and those who were retired (unable to work) had higher odds of contracting COVID-19 (aOR: 2.22, 2.05, and 11.36, respectively) ( Table 1).

### Perceived risk of dying from COVID-19 and associated determinants

Of the total respondents, 22.4% (370/1,649) had a perceived risk of dying from COVID-19, which was classified as high. In the unadjusted analysis, the level of perceived risk was associated with country, age group, gender, monthly household income, religion, having hypertension, heart disease, and pulmonary disease, and knowing or not knowing someone in the immediate social environment who were or had been infected with COVID-19 to varying degrees ( Table 2). In the adjusted logistic regression, country, age group, gender, religion, type of job, pulmonary disease, knowing people in the immediate social environment who were or had been infected with COVID-19, had seen or read about individuals infected with COVID-19 on social media or TV, and type of occupation were associated with a perceived risk of dying from COVID-19 ( Table 2).

**Table 2.** Logistic regression showing factors associated with perceived risk of dying from COVID-19 if infected (n=1,649).

Variable	n (%)	High perceived risk	Unadjusted		Adjusted	
		n (%)	OR (95% CI)	p-value	OR (95% CI)	p-value
Country						
Pakistan *(R)*	263 (15.9)	65 (24.7)	1		1	
Brazil	107 (6.5)	25 (23.4)	0.93 (0.55 – 1.58)	0.784	0.88 (0.40 – 1.92)	0.745
Chile	106 (6.4)	16 (15.1)	0.54 (0.30 – 0.99)	0.045	0.70 (0.30 – 1.60)	0.392
Egypt	98 (6.0)	26 (26.5)	1.10 (0.65 – 1.87)	0.724	1.00 (0.56 – 1.80)	0.990
India	339 (20.6)	56 (16.5)	0.60 (0.40 – 0.90)	0.013	0.93 (0.49 – 1.78)	0.823
Iran	141 (8.6)	32 (22.7)	0.89 (0.55 – 1.45)	0.651	0.63 (0.35 – 1.13)	0.117
Nigeria	161 (9.8)	25 (15.5)	0.56 (0.34 – 0.93)	0.026	0.83 (0.40 – 1.73)	0.622
Bangladesh	131 (7.9)	47 (35.9)	1.70 (1.08 – 2.68)	0.021	2.10 (1.23 – 3.58)	0.006
Sudan	174 (10.6)	44 (25.3)	1.03 (0.66 – 1.60)	0.892	0.74 (0.44 – 1.25)	0.260
Tunisia	129 (7.8)	34 (26.4)	1.09 (0.67 – 1.77)	0.725	0.80 (0.45 – 1.44)	0.462
Age group (year)						
<20 *(R)*	280 (17.0)	54 (19.3)	1		1	
21-30	926 (56.2)	207 (22.4)	1.21 (0.86 – 1.68)	0.275	1.23 (0.83 – 1.85)	0.305
31-40	270 (16.4)	68 (25.2)	1.41 (0.94 – 2.11)	0.097	1.57 (0.90 – 2.75)	0.116
41-50	119 (7.2)	22 (18.5)	0.95 (0.55 – 1.65)	0.853	1.20 (0.60 – 2.40)	0.613
>51	54 (3.3)	19 (35.2)	2.27 (1.21 – 4.28)	0.011	2.70 (1.21 – 5.99)	0.015
Gender						
Male *(R)*	692 (42.0)	137 (19.8)	1		1	
Female	957 (58.0)	233 (24.3)	1.30 (1.03 – 1.65)	0.029	1.58 (1.20 – 2.08)	0.001
Urbanicity						
Rural *(R)*	314 (19.0)	70 (22.3)	1			
Urban	1335 (81.0)	300 (22.5)	1.01 (0.75 – 1.36)	0.945		
Monthly household income (USD)						
<500 *(R)*	618 (37.5)	167 (27.0)	1		1	
500-999	289 (17.5)	66 (22.8)	0.80 (0.58 – 1.11)	0.179	0.80 (0.56 – 1.14)	0.212
1,000-1,999	192 (11.6)	36 (18.8)	0.62 (0.42 – 0.93)	0.022	0.58 (0.38 – 0.90)	0.015
2,000-2,999	148 (9.0)	36 (24.3)	0.87 (0.57 – 1.32)	0.504	0.94 (0.59 – 1.49)	0.777
3,000-4,999	128 (7.8)	23 (18.0)	0.59 (0.36 – 0.96)	0.034	0.62 (0.36 – 1.05)	0.076
5,000-7,999	100 (6.1)	21 (21.0)	0.72 (0.43 – 1.20)	0.205	0.73 (0.42 – 1.28)	0.267
≥8,000	174 (10.6)	21 (12.1)	0.37 (0.23 – 0.61)	<0.001	0.34 (0.19 – 0.60)	<0.001
Religion						
Islam *(R)*	915 (55.5)	242 (26.4)	1		1	
Christian/Protestant/Methodist/Lutheran/Baptist	179 (10.9)	24 (13.4)	0.43 (0.27 – 0.68)	<0.001	0.54 (0.29 – 1.00)	0.051
Catholic	127 (7.7)	28 (22.0)	0.79 (0.50 – 1.23)	0.290	0.96 (0.50 – 1.85)	0.902
Hindu	239 (14.5)	32 (13.4)	0.43 (0.29 – 0.64)	<0.001	0.44 (0.24 – 0.81)	0.008
Atheist or agnostic	87 (5.3)	18 (20.7)	0.73 (0.42 – 1.24)	0.243	0.93 (0.47 – 1.84)	0.840
Others	102 (6.2)	26 (25.5)	0.95 (0.60 – 1.52)	0.835	1.05 (0.58 – 1.90)	0.862
Healthcare-related job						
No *(R)*	908 (55.1)	198 (21.8)	1			
Yes	741 (44.9)	172 (23.2)	1.08 (0.86 – 1.37)	0.496		
Have hypertension						
No ^a^ *(R)*	1102 (66.8)	227 (20.6)	1		1	
Yes ^b^	97 (5.9)	32 (33.0)	1.90 (1.21 – 2.97)	0.005	1.29 (0.75 – 2.22)	0.365
Do not know	450 (27.3)	111 (24.7)	1.26 (0.97 – 1.64)	0.078	1.38 (0.96 – 1.99)	0.087
Have diabetes						
No ^a^ *(R)*	1190 (72.2)	266 (22.4)	1			
Yes ^b^	58 (3.5)	16 (27.6)	1.32 (0.73 – 2.39)	0.353		
Do not know	401 (24.3)	88 (21.9)	0.98 (0.74 – 1.28)	0.865		
Have heart disease						
No ^a^ *(R)*	1093 (66.3)	233 (21.3)	1		1	
Yes ^b^	55 (3.3)	24 (43.6)	2.86 (1.65 – 4.96)	<0.001	2.35 (1.20 – 4.60)	0.013
Do not know	501 (30.4)	113 (22.6)	1.08 (0.83 – 1.39)	0.578	1.03 (0.67 – 1.58)	0.901
Have pulmonary disease						
No ^a^ *(R)*	1044 (63.3)	215 (20.6)	1		1	
Yes ^b^	90 (5.5)	42 (46.7)	3.37 (2.17 – 5.24)	<0.001	2.88 (1.73 – 4.78)	<0.001
Do not know	515 (31.2)	113 (21.9)	1.08 (0.84 – 1.40)	0.539	0.88 (0.57 – 1.37)	0.578
Know people in immediate social environment who are or have been infected with COVID-19						
No *(R)*	508 (30.8)	93 (18.3)	1		1	
Yes	1141 (69.2)	277 (24.3)	1.43 (1.10 – 1.86)	0.007	1.51 (1.11 – 2.05)	0.008
Have you seen or read about individuals infected with the COVID-19 on social media or TV?						
No *(R)*	121 (7.3)	33 (27.3)	1		1	
Yes	1528 (92.7)	337 (22.1)	0.76 (0.50 – 1.15)	0.187	0.70 (0.44 – 1.10)	0.122
Occupation						
Self-employed *(R)*	155 (9.4)	35 (22.6)	1		1	
Employed for wages	417 (25.3)	103 (24.7)	1.13 (0.73 – 1.74)	0.599	1.12 (0.69 – 1.81)	0.643
Out of work for less 1 year AND more than 1 year	73 (4.4)	23 (31.5)	1.58 (0.85 – 2.94)	0.150	1.64(0.83 – 3.25)	0.153
Homemaker	34 (2.1)	12 (35.3)	1.87 (0.84 – 4.15)	0.124	1.52 (0.63 – 3.68)	0.351
Student	948 (57.5)	192 (20.3)	0.87 (0.58 – 1.31)	0.507	0.92 (0.56 – 1.49)	0.720
Retired or unable to work	22 (1.3)	5 (22.7)	1.01 (0.35 – 2.93)	0.988	0.70 (0.21 – 2.31)	0.558
Has how much your work changed as a result of the COVID-19 pandemic?						
No change or not applicable (not working) *(R)*	1009 (61.2)	203 (20.1)	1		1	
I work more hours	300 (18.2)	80 (26.7)	1.44 (1.07 – 1.95)	0.016	1.39 (0.99 – 1.96)	0.056
I work fewer hours	286 (17.3)	71 (24.8)	1.31 (0.96 – 1.79)	0.086	1.25 (0.89 – 1.75)	0.192
I was let go from my job	54 (3.3)	16 (29.6)	1.67 (0.91 – 3.06)	0.095	1.26 (0.65 – 2.46)	0.497

COVID-19, coronavirus disease 2019; OR, odds ratio; CI, confidence interval; R, reference group.

^a^ Have been tested or examined by a doctor but negative.

^b^ Have been diagnosed by a doctor.

Our data suggested that the elderly group (those over the age of 51) had a higher perceived risk of dying from COVID-19, if infected, by 2.7 times (95% CI: 1.21–5.99) compared to the youngest age group (those under the age of 20) ( Table 2). Compared to male participants, female participants had 1.5 times higher odds of perceived risk of dying from COVID-19 (aOR: 1.58; 95% CI: 1.20–2.08, p=0.001). Participants who had heart disease and pulmonary disease had a 2.3- and 2.8-fold chance of having a perceived risk of dying from COVID-19 compared to those who did not have such comorbidities (aOR: 2.35; 95% CI: 1.20–4.60 and aOR: 2.88; 95% CI: 1.73–4.78, respectively). Our study also found that participants who knew someone from their immediate social environment who were or had been infected with COVID-19 had higher odds of perceiving the risk of dying from COVID-19 with an aOR: 1.51; 95% CI: 1.11–2.05.

## Discussion

An individual’s perception of COVID-19-associated risks plays a major role in the ongoing pandemic. ^12^ At the community level, perceived risk contributes to increased public participation in preventive measures devised by governments and other health organizations. In addition, it encourages voluntary health behaviors that are beneficial in controlling the spread of SARC-CoV-2. ^12^^,^^13^

According to our findings, 36.4% of participants had a high perceived risk of COVID-19 infection, while only 22.4% had a high perceived risk of dying from COVID-19. This is lower compared to the other studies. ^14^^–^^16^ In a cross-sectional study conducted in Myanmar, the COVID-19-associated risk perception level was reported to be moderate to high. ^16^ However, a study conducted in Hong Kong reported high levels of perceived risk among participants. ^17^ In another study, higher risk perception was found to be associated with spending more time at home. Furthermore, higher risk perception was also observed in individuals living in counties with higher mortality rates. ^12^ Therefore, governments and other health agencies should focus on increasing the COVID-19 risk perception of the public to enhance voluntary health behaviors. Our study found that female participants had a higher perceived risk of being infected and dying from COVID-19 compared to their male counterparts. Several studies have reported similar findings. Men are less concerned about the potential health consequences associated with COVID-19 and, therefore, express a lower perceived risk of infection. ^18^

In an online survey conducted in Ethiopia, perceived risk was found to be dependent on age, occupation, educational status, and place of residence. ^14^ Our study found that participants working in healthcare-related sectors had a higher perceived risk of contracting COVID-19 compared to those working in non-healthcare sectors. Previous studies have reported that the perceived risk of becoming infected is higher among healthcare professionals. ^19^ The high perceived risk among individuals working in healthcare-related sectors can be attributed to their better knowledge of COVID-19 than the general population. ^20^ In addition, being a woman, having pulmonary disease, knowing people in the immediate social environment who were or have been infected with COVID-19, and seeing or reading about individuals infected with COVID-19 on social media or TV were also associated with a higher perceived risk of being infected with COVID-19.

It has been already established that individuals with chronic diseases (such as heart diseases, chronic obstructive pulmonary disease, hypertension, diabetes, and cancer) are at an increased risk for adverse outcomes with COVID-19. ^13^^,^^21^ In our study, individuals with pulmonary disease had a higher perceived risk of COVID-19 infection than healthy individuals. Furthermore, knowing people in the immediate social environment who were or have been infected with COVID-19, as well as seeing or reading about individuals infected with COVID-19 on social media or TV, were associated with a higher perceived risk. Similarly, being an elderly individual with heart disease and pulmonary disease, knowing people in the immediate social environment who were or had been infected with COVID-19, as well as seeing or reading about individuals infected with COVID-19 on social media or TV, also had a higher perceived risk of death due to COVID-19.

Being an online survey, there are a few limitations that can lead to bias in this study. For example, this survey might have excluded individuals belonging to lower socioeconomic classes, those with lower educational qualifications, and those who are illiterate. In addition, variation in Internet access might have contributed to selection bias across the countries evaluated in this study. Furthermore, the participants may also respond in a manner that may cause a social desirability bias.

## Conclusions

Our data suggests that there is a low perceived risk of becoming infected and dying due to COVID-19 among community members in certain LMICs. Factors such as age group, sex, religion, type of job, health status, etc., modify the perceived risk. These determinants can be used to alter and influence community health behaviors. Governments and other health agencies can use the data from this study to target susceptible groups within the community by conducting health campaigns to disseminate knowledge and information on the ongoing pandemic.

## Data availability

### Underlying data

Figshare: Perceived risk of infection and death from COVID-19 among community members of low- and middle-income countries. https://doi.org/10.6084/m9.figshare.19128134. ^22^

The project contains the following underlying data:

- Perceived Risk Master Data.xlsx
- Questionnaire of Perceived risk of infection and death from COVID-19 among community members of low- and middle-income countries.pdf

### Reporting guidelines

Figshare: STROBE checklist for ‘Perceived risk of infection and death from COVID-19 among community members of low- and middle-income countries’. https://doi.org/10.6084/m9.figshare.19128332. ^23^

Data are available under the terms of the Creative Commons Attribution 4.0 International license (CC-BY 4.0).
