# Supplementary material for: Perceived risk of infection and death from COVID-19 among community members of low- and middle-income countries: A cross-sectional study
Source: F1000Res. 2022 Sep 7;11:345. Originally published 2022 Mar 22. [Version 2] doi: 10.12688/f1000research.109575.2 (PMC9468621; doi:10.12688/f1000research.109575.2)
Supplement: Peer review history

# Peer review: Reviewer response for version 2

**DOI:** 10.5256/f1000research.138139.r149780

**Published:** 2022-09-12

**Authors:** Hesham M Al-Mekhlafi

**Affiliations:** 1 Medical Research Centre, Jazan University, Jazan, Saudi Arabia; 2 Department of Parasitology, Faculty of Medicine and Health Sciences, Sana’a University, Sana’a, Yemen

The authors have adequately addressed my comments.

Is the work clearly and accurately presented and does it cite the current literature?

Yes

If applicable, is the statistical analysis and its interpretation appropriate?

Yes

Are all the source data underlying the results available to ensure full reproducibility?

Yes

Is the study design appropriate and is the work technically sound?

Yes

Are the conclusions drawn adequately supported by the results?

Yes

Are sufficient details of methods and analysis provided to allow replication by others?

Partly

Reviewer Expertise:

Infectious Diseasis, Epidemiology, Public Health, Biostatistics

I confirm that I have read this submission and believe that I have an appropriate level of expertise to confirm that it is of an acceptable scientific standard.

# Peer review: Reviewer response for version 2

**DOI:** 10.5256/f1000research.138139.r149781

**Published:** 2022-09-08

**Authors:** Erwin Astha Triyono

**Affiliations:** 1 Department of Internal Medicine, Faculty of Medicine, Universitas Airlangga, Surabaya, Indonesia

Thanks for your response. Revised article is enough.

Is the work clearly and accurately presented and does it cite the current literature?

Yes

If applicable, is the statistical analysis and its interpretation appropriate?

Yes

Are all the source data underlying the results available to ensure full reproducibility?

Yes

Is the study design appropriate and is the work technically sound?

Yes

Are the conclusions drawn adequately supported by the results?

Yes

Are sufficient details of methods and analysis provided to allow replication by others?

Yes

Reviewer Expertise:

Tropical and Infectious Disease, Internal Medicine, Problem Health

I confirm that I have read this submission and believe that I have an appropriate level of expertise to confirm that it is of an acceptable scientific standard.

# Peer review: Reviewer response for version 1

**DOI:** 10.5256/f1000research.121089.r146559

**Published:** 2022-08-09

**Authors:** Erwin Astha Triyono

**Affiliations:** 1 Department of Internal Medicine, Faculty of Medicine, Universitas Airlangga, Surabaya, Indonesia

A well-written article by collecting data taken from an online survey of several countries to analyze the perceived risk of infection and death due to Covid-19. Overall, this article is interesting and provides many references for its readers. What I learned from this article is that Covid-19 poses challenges to a country's health system. These challenges can be related to medical problems and non-medical problems (social problems). Non-medical problems (social problems) arise due to disinformation or hoax issues that make it difficult for medical/scientific information to be conveyed to the public in its entirety. This researcher is very helpful in describing the obstacles, especially in the non-medical aspect (social problems) in handling Covid-19 in a country.

 However, there are some comments for improvement:

1. Need to explain the sampling method used, how to determine 10 countries?
2. The researcher explained that the limitations of the survey conducted could bias the results, especially because of the possibility that this survey might have excluded individuals belonging to lower socioeconomic classes, those with lower educational qualifications, and those who are illiterate. Can it be written in the conclusion to be a suggestion for further research?

Is the work clearly and accurately presented and does it cite the current literature?

Yes

If applicable, is the statistical analysis and its interpretation appropriate?

Yes

Are all the source data underlying the results available to ensure full reproducibility?

Yes

Is the study design appropriate and is the work technically sound?

Yes

Are the conclusions drawn adequately supported by the results?

Yes

Are sufficient details of methods and analysis provided to allow replication by others?

Yes

Reviewer Expertise:

Tropical and Infectious Disease, Internal Medicine, Problem Health

I confirm that I have read this submission and believe that I have an appropriate level of expertise to confirm that it is of an acceptable scientific standard.

# Response

**Published:** 2022-08-31

**Authors:** Harapan Harapan

**Affiliations:** Syiah Kuala University, Indonesia

Dear reviewer,

 Thank you for reviewing our manuscript. The comments and suggestions have improved the quality of our current manuscript significantly.

 1. Need to explain the sampling method used, how to determine 10 countries?

 RESPONSE: Thank you for the question. Those countries were selected to cover of those in LMICs in three continents that had limited information of COVID-19. Also based on the availability of the collaborators.

 Information about the sampling technique. In the revised manuscript: “This study used nonprobability sampling technique, snowball sampling method, to recruit the respondents. The invited potential respondents were also requested to share the invitation to their friends or phone contacts.”

 2. The researcher explained that the limitations of the survey conducted could bias the results, especially because of the possibility that this survey might have excluded individuals belonging to lower socioeconomic classes, those with lower educational qualifications, and those who are illiterate. Can it be written in the conclusion to be a suggestion for further research?

 RESPONSE: Thank you for the suggestion. However, we feel this is not part of the conclusion of the study and we would like to provide the actual results of the study within the conclusion. 

 Thank you for the reviewing our manuscript.

# Peer review: Reviewer response for version 1

**DOI:** 10.5256/f1000research.121089.r130137

**Published:** 2022-05-24

**Authors:** Hesham M Al-Mekhlafi

**Affiliations:** 1 Medical Research Centre, Jazan University, Jazan, Saudi Arabia; 2 Department of Parasitology, Faculty of Medicine and Health Sciences, Sana’a University, Sana’a, Yemen

**Ref. No** **:** **F1KR00CDE F1R-VER121089-R**

**Title: Perceived risk of infection and death from COVID-19 among community members of low- and middle-income countries: A cross-sectional study**

 This manuscript describes a cross-sectional online survey on the perceived risk of contracting and dying from COVID-19 among 1,646 respondents from 10 LMICs. The study found relatively low levels of perceived risk of infection and death, and identified some demographic, socio-economic and health factors that significantly associated with high perceived levels. Although, the topic is not novel as many studies on the same topic are available from different countries; however, data at the regional or multinational level are always interesting and appreciated.

 Overall, the manuscript is interesting, well-written and straightforward. Some comments are provided below.

**SPECIFIC COMMENTS**

1. Study design and setting: What is the rationale of selecting the 10 countries included in the study? What was the selection strategy?
2. How was the selection for enrollment process? The questionnaire link was shared on Twitter, Facebook, and WhatsApp. More important information is needed about this process. How to ensure that the link has reached the targeted population? How WhatsApp numbers were selected, etc.?
3. Study design and setting: Other study design elements also need to be clearly described in the manuscript for a better understanding of the studied population and what they represent. For example, what is being considered a healthcare-related job in this study?
4. What is the rationale of choosing Pakistan as a reference group for country variable?
5. Page 5: “able to read and understand English in the 10 studied countries.” Could this be source of bias, in respect to the aim of helping in the design of population-wide preventive measures?
6. Page 13, discussion, 3 ^rd^ paragraph: working in non-healthcare sectors was the only variable explained while the rest were repeated as results. Why did females have higher perceived risk than males?
7. Page 13, discussion: Some significant variables were omitted in discussion and not explained, e.g. religion, type of occupation. What about country as a predictor? Any explanation?
8. Page 5: “which 203 respondents were excluded due to incomplete information.” An advantage of online questionnaire is that it can be designed as a respondent cannot proceed with incomplete responses!
9. Page 6: “65% were part of a family with a monthly household income of less than $2,000”. The tables show that 37.5% of the respondents had a monthly household income of less than $500. I think the later is better representative the studied population and it should be indicated.   
10. Page 6, Results, Perceived risk of becoming infected with COVID-19 and the associated determinants, first paragraph: The variables found significant by the unadjusted model were also found significant in the adjusted model. The sentences can be rephrased without repeating the list of variables, especially with the variables and related statistics are mentioned in the next paragraph. Gender was found significant only in the adjusted model and this can be indicated.
11. Urbanicity or residency?
12. Page 6, results: “…… 22.4% (370/1,649) had a perceived risk of dying from COVID-19, which was classified as high”. This can be rephrased to avoid confusion with a statement in page 3, conclusion “are relatively low”.
13. Page 6, results: “….. knowing or not knowing someone …”, please rephrase.
14. Page 6, results, perceived risk of becoming infected…: again, the sentences can be rephrased without repeating the list of variables, especially with the variables and related statistics are mentioned in the next paragraph in page 13.
15. Page 13, discussion: “This is lower compared to the other studies”. Before this comparison, it should be indicated that the overall reported rates were relatively low.

Is the work clearly and accurately presented and does it cite the current literature?

Yes

If applicable, is the statistical analysis and its interpretation appropriate?

Yes

Are all the source data underlying the results available to ensure full reproducibility?

Yes

Is the study design appropriate and is the work technically sound?

Yes

Are the conclusions drawn adequately supported by the results?

Yes

Are sufficient details of methods and analysis provided to allow replication by others?

Partly

Reviewer Expertise:

Infectious Diseasis, Epidemiology, Public Health, Biostatistics

I confirm that I have read this submission and believe that I have an appropriate level of expertise to confirm that it is of an acceptable scientific standard, however I have significant reservations, as outlined above.

# Response

**Published:** 2022-08-31

**Authors:** Harapan Harapan

**Affiliations:** Syiah Kuala University, Indonesia

Dear reviewer,

 Thank you for reviewing our manuscript. The comments and suggestions have improved the quality of our current manuscript significantly.

 1. Study design and setting: What is the rationale of selecting the 10 countries included in the study? What was the selection strategy?

 RESPONSE: Thank you for the question. Those countries were selected to cover of those in LMICs in three continents that had limited information of COVID-19. Also based on the availability of the collaborators. 

 2. How was the selection for enrollment process? The questionnaire link was shared on Twitter, Facebook, and WhatsApp. More important information is needed about this process. How to ensure that the link has reached the targeted population? How WhatsApp numbers were selected, etc.?

 RESPONSE: Thank you we have provided the information about the sampling technique. In the revised manuscript: “This study used nonprobability sampling technique, snowball sampling method, to recruit the respondents. The invited potential respondents were also requested to share the invitation to their friends or phone contacts.” 

 Due to the this in the Discussion we discussed this as limitation of our study. “…this survey might have excluded individuals belonging to lower socioeconomic classes, those with lower educational qualifications, and those who are illiterate”. 

 3.Study design and setting: Other study design elements also need to be clearly described in the manuscript for a better understanding of the studied population and what they represent. For example, what is being considered a healthcare-related job in this study?

 RESPONSE: We have explained in some parts of the Study design and setting. The explanation of healthcare-related job has been provided under sub-heading “Explanatory variables”. We stated that “Healthcare-related job included doctor, dentist, nurse, physical therapist, nutritionist, pharmacist, paramedic, and laboratory staff.” 

 4. What is the rationale of choosing Pakistan as a reference group for country variable?

 RESPONSE: Pakistan was chosen after the initial analyzing showing Pakistan had the lowest perceived risk rate. 

 5. Page 5: “able to read and understand English in the 10 studied countries.” Could this be source of bias, in respect to the aim of helping in the design of population-wide preventive measures?

 RESPONSE: We appreciate this. We acknowledged for this potential bias and we have included this as one of limitations of our study. “In addition, since the survey was conducted in English, this could be source of bias because some of the individuals in those studied countries might unable to read and understand English.”

 6. Page 13, discussion, 3rd paragraph: working in non-healthcare sectors was the only variable explained while the rest were repeated as results. Why did females have higher perceived risk than males?

 RESPONSE: We have explained some explanatory variable. However, we focused to discuss the variables to determine the groups that could be targeted.

 In our article, we provided the possible explanation why the females had higher perceived risk: “Men are less concerned about the potential health consequences associated with COVID-19 and, therefore, express a lower perceived risk of infection”.

 7. Page 13, discussion: Some significant variables were omitted in discussion and not explained, e.g. religion, type of occupation. What about country as a predictor? Any explanation?

 RESPONSE: Thank you for you question. We aim to provide general overview in Discussion not too detailed and discussed the factors to determine the specific groups that are able to targeted to increase the perceived risk in the future. We have discussed the factors associated with perceived risk such as gender, type of occupation, having the comorbid and soon in the Discussion. 

 8. Page 5: “which 203 respondents were excluded due to incomplete information.” An advantage of online questionnaire is that it can be designed as a respondent cannot proceed with incomplete responses!

 RESPONSE: Thank you. The survey has been set that all question should be answer to be able to go to the next questions. However, some respondents stopped the survey in the middle of the survey, of which this was classified as incomplete responses. 

 9. Page 6: “65% were part of a family with a monthly household income of less than $2,000”. The tables show that 37.5% of the respondents had a monthly household income of less than $500. I think the later is better representative the studied population and it should be indicated.

 RESPONSE: I think both of the statements are correct. We would like not to repeat what in the table and text. However, the we have revised the text based on the recommendation. In the revised manuscript: "The majority (81%) of the respondents lived in urban areas, and 37.5% were part of a family with a monthly household income of less than $500."**

 10. Page 6, Results, Perceived risk of becoming infected with COVID-19 and the associated determinants, first paragraph: The variables found significant by the unadjusted model were also found significant in the adjusted model. The sentences can be rephrased without repeating the list of variables, especially with the variables and related statistics are mentioned in the next paragraph. Gender was found significant only in the adjusted model and this can be indicated.

 RESPONSE: We have revised this section. We would like to thank you for the suggestion. The revised manuscript: “The unadjusted and adjusted logistic regression suggested that the country, age group, religion, type of job, having pulmonary disease, knowing people in the immediate social environment who were or had been infected with COVID-19, seeing or reading about individuals infected with COVID-19 on social media or TV, and type of occupation were associated with a perceived risk of contracting COVID-19 (Table 1). Gender was only associated significantly with perceived risk in adjusted logistic regression.”

 11. Urbanicity or residency?

 RESPONSE: Thank you for your suggestion. Now it has been changed to residency. 

 12. Page 6, results: “…… 22.4% (370/1,649) had a perceived risk of dying from COVID-19, which was classified as high”. This can be rephrased to avoid confusion with a statement in page 3, conclusion “are relatively low”.

 RESPONSE: We have revised the manuscript based on the suggestion. The revised manuscript: “Of the total respondents, only 22.4% (370/1,649) had an adequate (high) perceived risk of dying from COVID-19.”

 13. Page 6, results: “….. knowing or not knowing someone …”, please rephrase.

 RESPONSE: This has been corrected: “In the unadjusted analysis, the level of perceived risk was associated with country, age group, gender, monthly household income, religion, having hypertension, heart disease, and pulmonary disease, *and knowing someone in the immediate social environment who were* or had been infected with COVID-19 to varying degrees (Table 2).”

 14. Page 6, results, perceived risk of becoming infected…: again, the sentences can be rephrased without repeating the list of variables, especially with the variables and related statistics are mentioned in the next paragraph in page 13.

 RESPONSE: We would like to thank you for this. We have corrected this section by providing the adjusted logistic regression only. In the revised manuscript: “In the adjusted logistic regression, country, age group, gender, religion, type of job, having pulmonary disease, knowing people in the immediate social environment who were or had been infected with COVID-19, had seen or read about individuals infected with COVID-19 on social media or TV, and type of occupation were associated with a perceived risk of dying from COVID-19 (Table 2).”

 15. Page 13, discussion: “This is lower compared to the other studies”. Before thiscomparison, it should be indicated that the overall reported rates were relatively low.

 RESPONSE: Thank you. We have revised the text to provide the clear information. In the revised manuscript: “According to our findings, the percentages of participants who had adequate perceived risk of COVID-19 infection and perceived risk of dying from COVID-19 were relatively low, only 36.4% and 22.4%, respectively. These are lower compared to the other studies. ^14 –^ ^16”^

^Thank you^
